# The neuroprotective effects of FG-4592, a hypoxia-inducible factor-prolyl hydroxylase inhibitor, against oxidative stress induced by alpha-synuclein in N2a cells

**DOI:** 10.1038/s41598-023-42903-7

**Published:** 2023-09-20

**Authors:** Ayaka Fujimaki, Kazuki Ohuchi, Shinnosuke Takizawa, Takanori Murakami, Hisaka Kurita, Isao Hozumi, Xiaopeng Wen, Yoshihisa Kitamura, Zhiliang Wu, Yoichi Maekawa, Masatoshi Inden

**Affiliations:** 1 Laboratory of Medical Therapeutics and Molecular Therapeutics, Gifu Pharmaceutical University, Gifu, 501-1196 Japan; 2 Laboratory of Pharmacology and Neurobiology, College of Pharmaceutical Sciences, Ritsumeikan University, Shiga, 525-8577 Japan; 3 Department of Parasitology and Infectious Diseases, Gifu University Graduate School of Medicine, Gifu, 501-1194 Japan; 4 Division of Preemptive Food Research, Preemptive Food Research Center (PFRC), Gifu University Institute for Advanced Science (GUIAS), Gifu, 501-1194 Japan; 5 Division of Animal Medical Science, Center for One Medicine Innovative Translational Research (COMIT), Gifu University Institute for Advanced Science (GUIAS), Gifu, 501-1194 Japan

**Keywords:** Neurodegenerative diseases, Parkinson's disease

## Abstract

Parkinson’s disease (PD) is a neurodegenerative disorder characterized by the loss of dopaminergic neurons in the substantia nigra. The pathological hallmark of PD is the appearance of intraneuronal cytoplasmic α-synuclein (α-Syn) aggregation, called Lewy bodies. α-Syn aggregation is deeply involved in the pathogenesis of PD. Oxidative stress is also associated with the progression of PD. In the present study, to investigate whether a hypoxia-inducible factor (HIF)-prolyl hydroxylase (PH) inhibitor, FG-4592 (also called roxadustat), has neuroprotective effects against α-Syn-induced neurotoxicity, we employed a novel α-Syn stably expressing cell line (named α-Syn-N2a cells) utilizing a piggyBac transposon system. In α-Syn-N2a cells, oxidative stress and cell death were induced by α-Syn, and FG-4592 showed significant protection against this neurotoxicity. However, FG-4592 did not affect α-Syn protein levels. FG-4592 triggered heme oxygenase-1 (HO-1) expression downstream of HIF-1α in a concentration-dependent manner. In addition, FG-4592 decreased the production of reactive oxygen species possibly via the activation of HO-1 and subsequently suppressed α-Syn-induced neurotoxicity. Moreover, FG-4592 regulated mitochondrial biogenesis and respiration via the induction of the peroxisome proliferator-activated receptor-γ coactivator-1α. As FG-4592 has various neuroprotective effects against α-Syn and is involved in drug repositioning, it may have novel therapeutic potential for PD.

## Introduction

Parkinson’s disease (PD) is an age-related neurodegenerative disease that is characterized by relatively selective nigrostriatal dopamine (DA) neurodegeneration and the presence of intraneuronal cytoplasmic inclusions called Lewy bodies, which are consistently immunostained with antibodies to α-synuclein (α-Syn). Clinically, patients often present tremors, rigidity, postural instability, and bradykinesia accompanied by nonmotor symptoms (such as fatigue, depression, and dementia)^1^. Most PD cases are sporadic, with unknown etiology. Approximately 5–10% of PD cases seem to have monogenic forms of inheritance, including autosomal dominant (e.g., *SNCA*, *LRRK2*, and *VPS35*) and recessive (e.g., *PARK2*, *PINK1*, *DJ1*) forms^2–4^. Although the precise mechanism underlying the onset of PD has not been validated, oxidative stress, excessive free-radical generation, environmental toxins, and/or genetic factors may be intricately involved in the massive loss of nigrostriatal DA neurons^5^. The fact that many of the currently used therapies are symptomatic and not disease-modifying underscores the need for therapies that target the key etiologic components of PD.

Hypoxia-inducible factor-1 (HIF-1) is a heterodimeric transcription factor that functions pivotally in regulating cellular oxygen homeostasis. It comprises an oxygen-regulated HIF-1α subunit and a constitutively expressed HIF-1β subunit. HIF-1α is hydroxylated by prolyl hydroxylase proteins (PHD) under normal levels of oxygen or reactive oxygen species (ROS), ubiquitinated by Von Hippel Lindau (VHL) protein, and finally degraded by the proteasome^6^. However, hypoxia or elevated levels of ROS inhibit PHD, which in turn inhibits VHL-mediated degradation of HIF-1α. Following this stabilization, the active HIF-1 heterodimer is formed and binds to the hypoxia-responsive element (HRE) in the regulatory regions of many genes, including heme oxygenase-1 (HO-1). Remarkably, HIF-1α expression and its downstream targets are downregulated in the substantia nigra par compacta (SNpc) of PD brains^7^. Conversely, an increase in HIF-1α and/or its targets is protective in various models of PD^8^. In addition, hypoxic events are associated with the development of neurodegenerative diseases, such as PD^9^. Thus, strategies to enhance HIF-1α with PHD inhibitors and other agents may be effective as a novel treatment for PD.

FG-4592, known as roxadustat, is a small-molecule stabilizer of HIF that inhibits PHD, and it has been approved as the first-in-class orally active drug for the treatment of renal anemia^10^. A previous study showed that in 1-methyl-4-phenyl-1,2,3,6-tetrahydropyridine (MPTP)-treated mice, FG-4592 protects against MPTP-induced loss of DA neurons of SNpc and attenuates behavioral impairments via HIF-1α signaling^11^. Since neither Lewy body-like inclusions nor α-Syn aggregation is generally found in the brains of MPTP-treated mice, it is challenging to examine the influence of FG-4592 on α-Syn-associated neurotoxicity^5^. Thus, the effect of FG-4592 on α-Syn-related neurotoxicity remains unexamined. Recently, we generated a new α-Syn stably expressing cell line (hereafter, named α-Syn-N2a cells) utilizing a piggyBac transposon system^12^. In the present study, we analyzed the neuroprotective effects of FG-4592 on α-Syn neurotoxicity in α-Syn-N2a cells.

## Results

### FG-4592 prevents the neurotoxicity of α-synuclein

**Figure 1:**
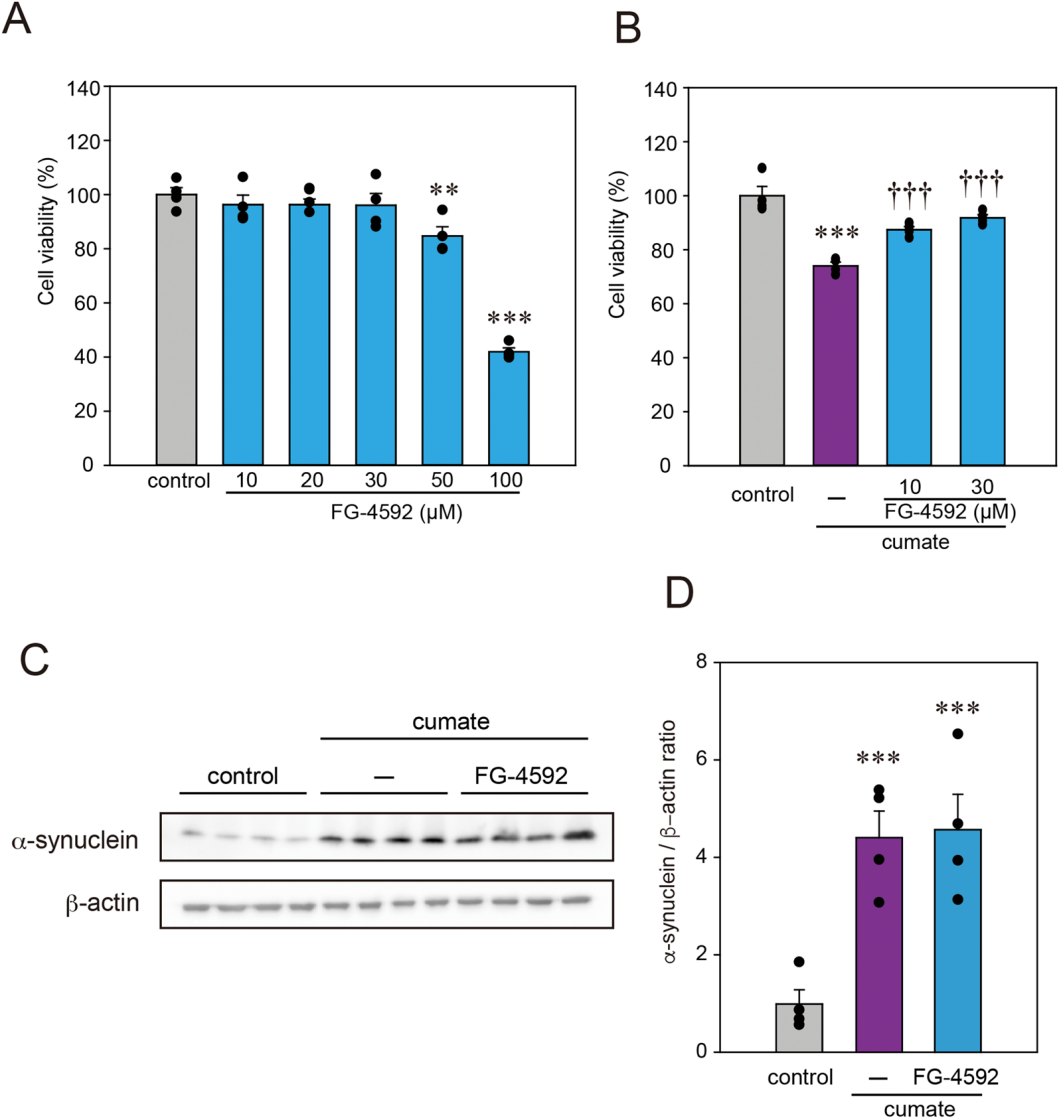
Effect of FG-4592 on cell viability and α-Syn protein levels in α-Syn-N2a cells. (**A**) α-Syn-N2a cells were treated with FG-4592 at different concentrations (10, 20, 30, 50, 100 μM) for 48 h. (**B**) α-Syn-N2a cells were treated with 50 µg/mL of cumate in the presence or absence of FG-4592 at various concentrations (10 and 30 μM) for 48 h. The cell viability was evaluated by CCK-8 assay. (**C**,**D**) α-Syn-N2a cells were treated with 50 µg/mL of cumate in the presence or absence of 30 µM FG-4592 for 48 h. Immunoblotting was used to examine the lysates using anti-α-synuclein and anti-β-actin antibodies. Normalized to β-actin expression, protein levels were assessed using the control band density. Data are denoted as mean ± SEM from four independent experiments. Significance: **p < 0.01, ***p < 0.001 vs. control, ^†††^p < 0.001 vs. cumate.

To analyze the effect of FG-4592 against α-Syn-related neurotoxicity, a piggyBac transposon system was used to stably express wild-type α-Syn in N2a cells in a cumate-inducible manner (α-Syn-N2a cells)^12^. First, the cytotoxicity of FG-4592 was validated in α-Syn-N2a cells using Cell Counting Kit-8 (CCK-8) assay. No cytotoxicity was detected when α-Syn-N2a cells were treated with 10–30 µM of FG-4592 for 48 h (Fig. 1A). In contrast, α-Syn-N2a cells treated with 50 and 100 µM of FG-4592 exhibited cell death. Hence, in subsequent experiments using α-Syn-N2a cells, 30 µM of FG-4592 was selected. As depicted in Fig. 1B, cell death was significantly stimulated with the induction of α-Syn via cumate treatment for 48 h, as reported in our previous study^12^. FG-4592 clearly inhibited α-Syn-induced neurotoxicity (Fig. 1B). Western blot analysis was performed in α-Syn-N2a cells to investigate the relationship between the protective effect of FG-4592 and the protein level of α-Syn (Fig. 1C,D). Surprisingly, FG-4592 did not affect the amount of α-Syn protein in α-Syn-N2a cells following exposure to cumate for 48 h. These results suggest that the neuroprotective effect of FG-4592 under our experimental conditions involves another protective effect that is indirectly linked to the amount of α-Syn protein.

### FG-4592 induces HO-1 protein expression via the HRE response pathway

**Figure 2:**
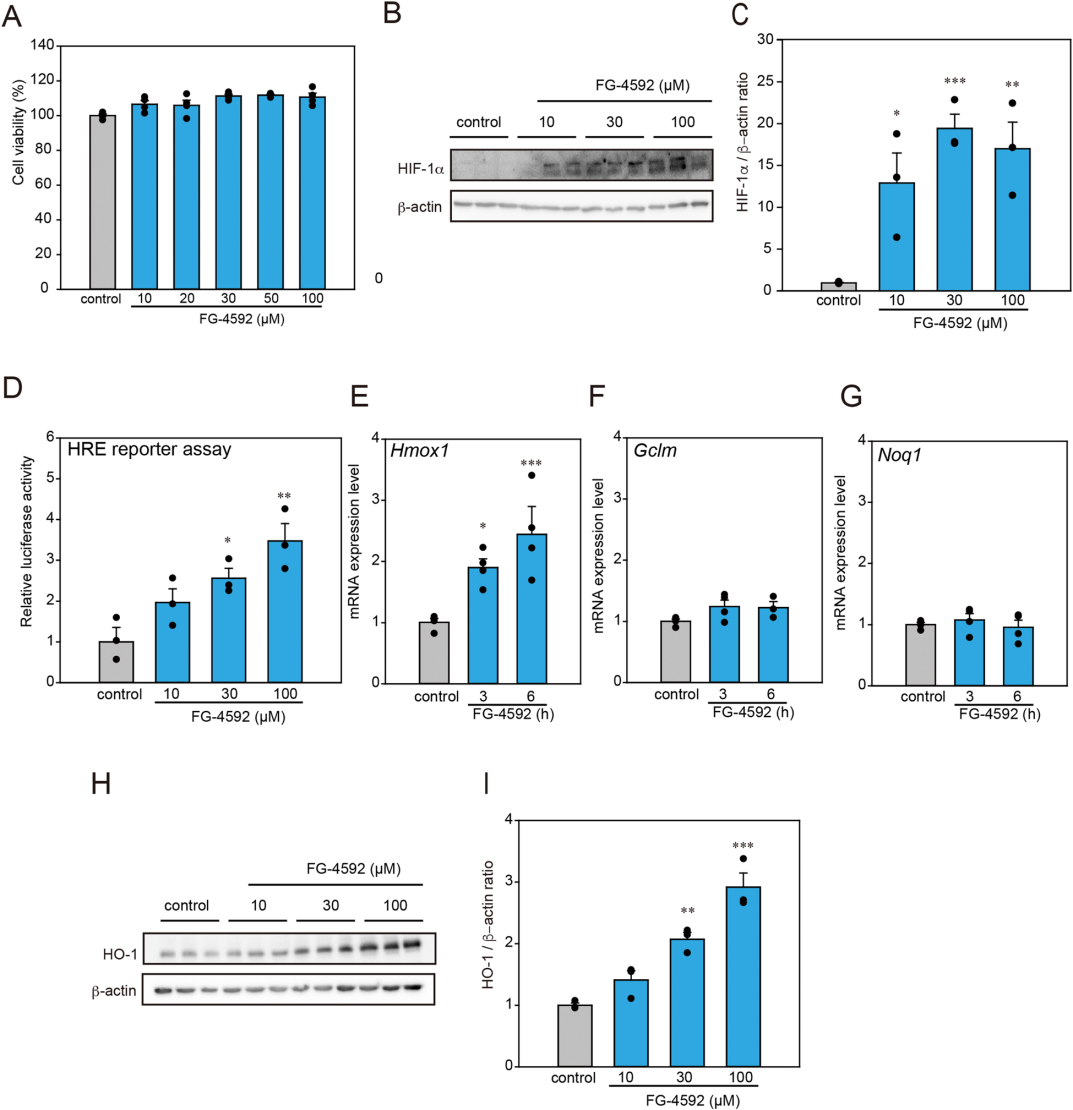
FG-4592 exerted the induction of HO-1. (**A**) N2a cells were treated with FG-4592 at various concentrations (10, 20, 30, 50, and 100 μM) for 48 h. (**B**,**C**) N2a cells were treated with FG-4592 at different concentrations (10, 30, 100 µM) for 12 h. The lysates were examined by immunoblotting with anti-HIF-1α and anti-β-actin antibodies. Protein levels assessed using the band density of the control and normalized to the expression of β-actin. (**D**) Results of HRE luciferase assays. (**E**–**G**) N2a cells were treated with 30 µM FG-4592 for 3 or 6 h, and mRNA expressions of *Hmox1* (**E**), *Gclm* (**F**), and *Nqo1* (**G**) were examined using the SYBR Green-based RT-qPCR assay. The expression levels of mRNA were normalized to the expression levels of *Gapdh* mRNA. (**H**,**I**) N2a cells were treated with FG-4592 at different concentrations (10, 30, 100 µM) for 24 h. The lysates were measured by immunoblotting with anti-HO-1 and anti-β-actin antibodies. Protein levels normalized to the expression of β-actin and quantified based on the band density of control. Data are represented as means ± SEM from three or four independent experiments. Significance: *p < 0.05, **p < 0.01, ***p < 0.001 vs. control.

To investigate the mechanism underlying the neuroprotective effect of FG-4592 against α-Syn-induced neurotoxicity, we focused on its effect on oxidative stress by upregulating HO-1, encoded by *Hmox1*, through the HIF-1α-HRE response pathway according to prior studies^6, 8, 11^. No cytotoxicity was detected when N2a cells were treated with 10–100 µM of FG-4592 for 24 h (Fig. 2A). Western blot analysis showed that FG-4592 increased HIF-1α protein levels (Fig. 2B,C). As the previous studies showed that HIF-1α was phosphorylated under stimulation such as hypoxia^13, 14^, the higher molecular band of HIF-1α protein in Fig. 2B could be phosphorylated HIF-1α (Fig. 2B). To analyze whether the actions of FG-4592 aid in the HRE response pathway, we conducted the reporter assay (Fig. 2D). The results revealed that FG-4592 induced luciferase activity in a dose-dependent manner, indicating that it contributes to the activity of the HRE reaction pathway by stabilizing HIF-1α. qRT-PCR analysis revealed that in N2a cells, *Hmox1* mRNA was increased by FG-4592 (Fig. 2E). However, other oxidative stress-related enzyme mRNAs, such as γ*-glutamyl cysteine synthetase modifier subunit* (*Gclm*) and *NAD(P)H: quinone oxidoreductase 1 (Nqo1)*, were unaffected in N2a cells (Fig. 2F,G). Western blot analysis also depicted that in N2a cells, the HO-1 protein was increased after FG-4592 treatment in a dose-dependent manner (Fig. 2H,I). These results suggest that HO-1 protein is induced by FG-4592 in N2a cells.

### FG-4592 reduces the production of ROS possibly via the induction of HO-1

**Figure 3:**
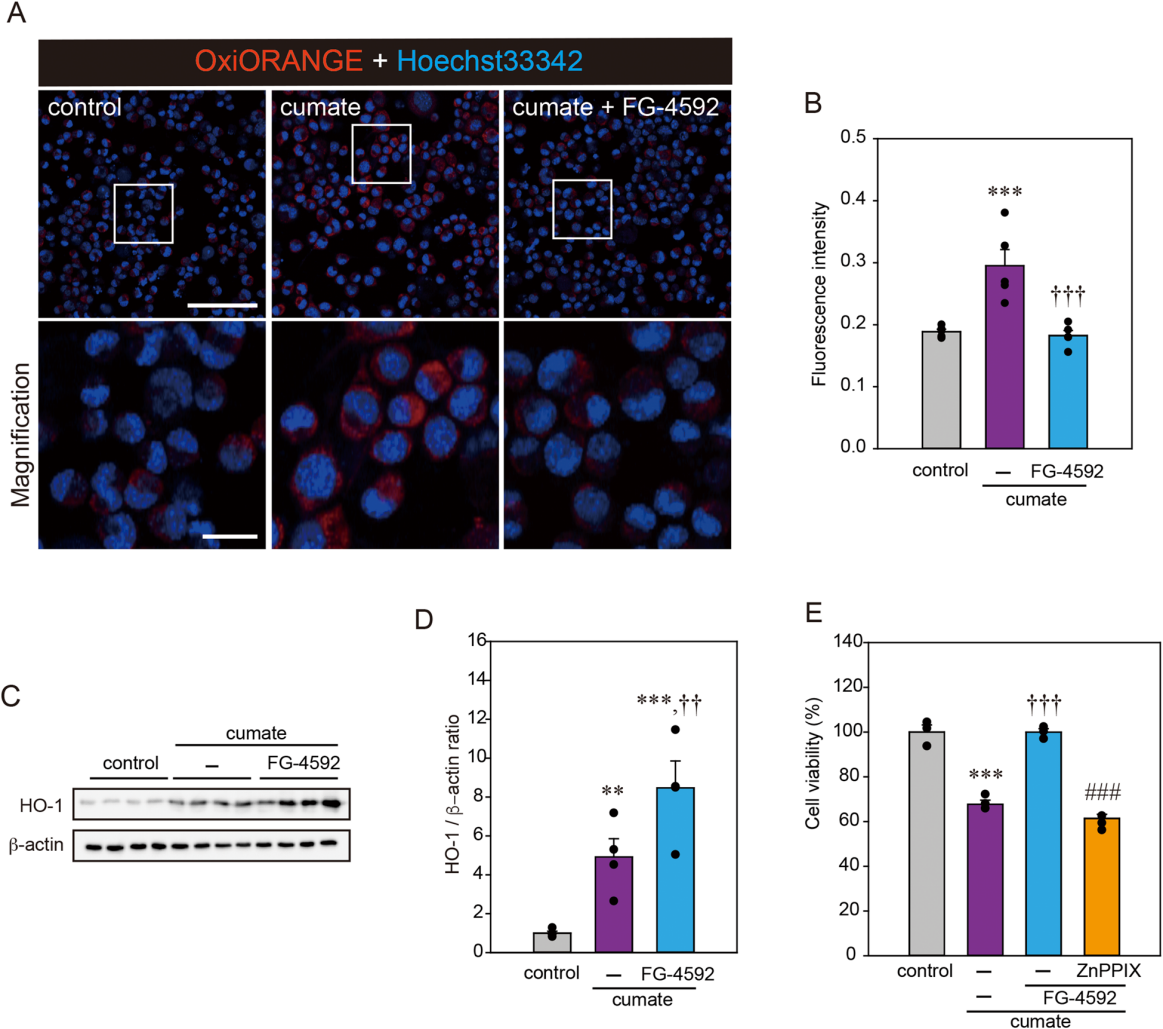
FG-4592 reduced oxidative stresses possibly via the induction of HO-1. (**A**,**B**) α-Syn-N2a cells were treated with 50 µg/mL of cumate in the presence or absence of 30 µM FG-4592 for 48 h. Fluorescence imaging was then conducted with OxiORANGE. (**A**) Representative images of OxiORANGE (scale bar, 100 µm) and magnified images (scale bar, 20 µm). (**B**) Quantified analysis of imaging. (**C**,**D**) α-Syn-N2a cells were treated with 50 µg/mL of cumate in the presence or absence of 30 µM FG-4592 for 48 h. Immunoblotting was used to evaluate the lysates using anti-HO-1 and anti-β-actin antibodies. Protein levels normalized to the expression of β-actin and quantified based on the band density of control. (**E**) α-Syn-N2a cells were treated with 50 µg/mL of cumate in the presence or absence of 30 µM FG-4592 and 3 µM Zinc protoporphyrin (ZnPPIX) for 72 h. Cell viability was estimated by a CCK-8 assay. Data are denoted as means ± SEM from four independent experiments. Significance: **p < 0.01, ***p < 0.001 vs. control, ^††^p < 0.01, ^†††^p < 0.001 vs. cumate, ^###^p < 0.001 vs. cumate and FG-4592.

Previous research demonstrated that mitochondrial dysfunction resulted from the α-Syn-induced production of ROS^15^. Therefore, we analyzed whether FG-4592 could affect the synthesis of ROS induced by α-Syn accumulation using OxiORANGE, a fluorogenic probe that measures oxidative stress^16^. Because of its positive charge, OxiORANGE localizes within the mitochondria. The fluorescence intensity in α-Syn-N2a cells displayed a marked increase when the cells were treated with cumate compared with the vehicle control. Treatment with FG-4592 significantly suppressed cumate-induced fluorescence (Fig. 3A,B). In addition, we investigated whether the actions of FG-4592 aid in the regulation of HO-1 protein levels in α-Syn-N2a cells (Fig. 3C,D). In α-Syn-N2a cells, HO-1 protein was significantly enhanced by cumate treatment, suggesting that the oxidative stress is caused by cumate-induced α-Syn. Simultaneous treatment with cumate and FG-4592 significantly improved HO-1 protein levels compared to treatment with cumate alone. Furthermore, to study whether the effects of HO-1 contribute to cell death triggered by cumate in α-Syn-N2a cells, we performed a CCK-8 assay with ZnPPIX as the HO-1 inhibitor. The protective effect of FG-4592 was significantly abolished by ZnPPIX treatment (Fig. 3E). These results suggest that FG-4592 lowers the production of ROS possibly via the induction of HO-1 and subsequently suppresses α-Syn-induced neurotoxicity.

### FG-4592 ameliorated α-Syn-induced mitochondrial dysfunction

**Figure 4:**
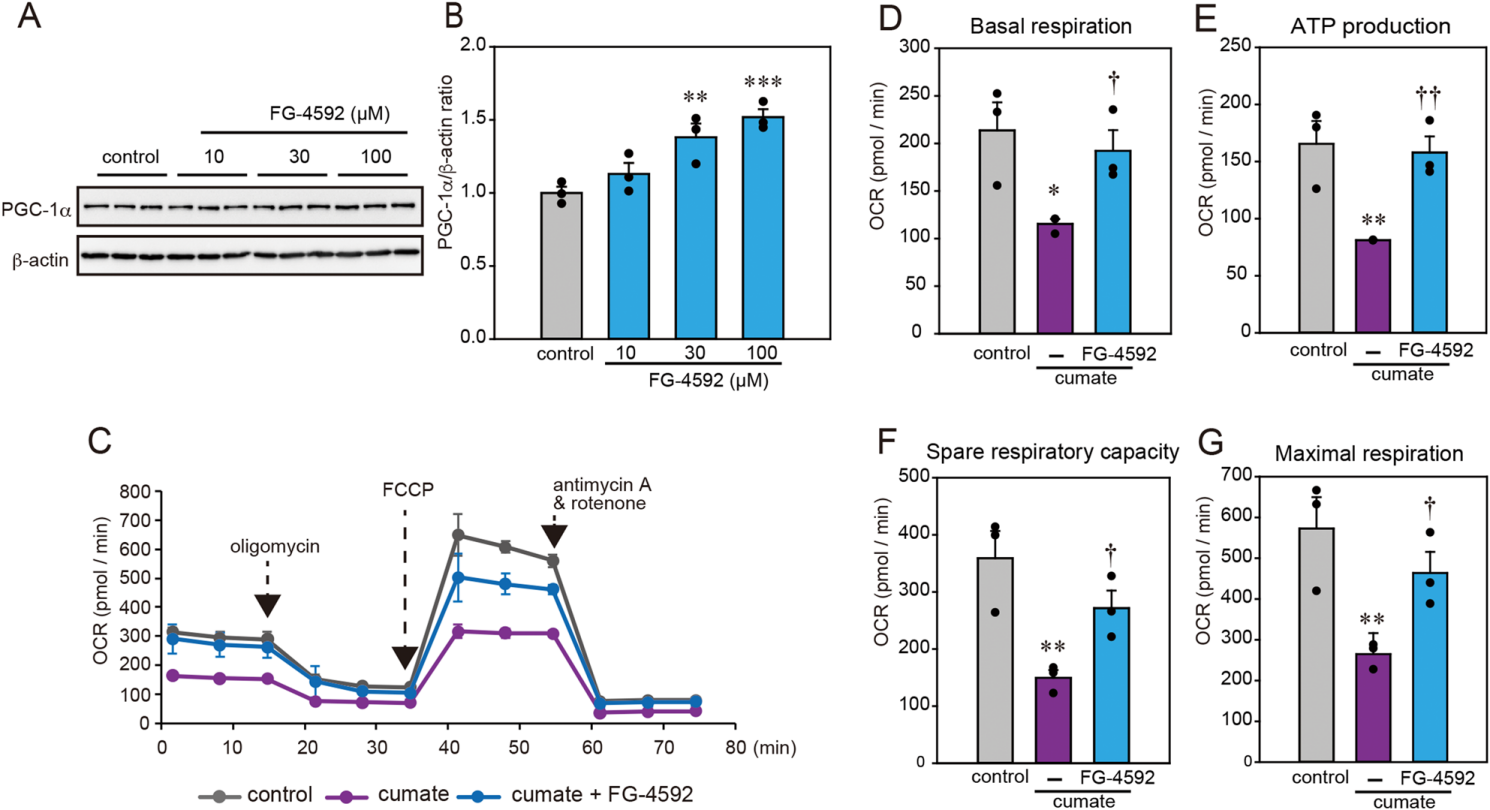
FG-4592 improved mitochondrial function. (**A**,**B**) N2a cells were treated with FG-4592 at different concentrations (10, 30, 100 µM) for 6 h. The lysates were examined by immunoblotting with anti-PGC-1α and anti-β-actin antibodies. Protein levels assessed using the band density of the control and normalized to the expression of β-actin. (**C**–**G**) α-Syn-N2a cells were treated with 50 µg/mL of cumate in the presence or absence of 30 µM FG-4592 for 48 h. Mitochondrial oxygen consumption rate (OCR) in α-Syn-N2a cells was assessed using Agilent Seahorse XF Cell Mito Stress Test Kit. We also list basal respiration (**D**), ATP production (**E**), maximal respiration (**F**), and spare respiratory capacity (**G**). Data are expressed as mean ± SEM from three independent experiments. Significance: *p < 0.05, **p < 0.01, ***p < 0.001 vs. control, ^†^p < 0.05, ^††^p < 0.01 vs. cumate.

Previous research demonstrated that FG-4592 increased the expression of peroxisome proliferator-activated receptor-γ coactivator-1α (PGC-1α), a key regulator of mitochondrial biogenesis and respiration, and also protected cells by resolving drug-induced mitochondrial dysfunction^11^. To examine whether the expression of PGC-1α was altered in N2a cells with FG-4592, we carried out an immunoblotting assay. Thus, FG-4592 increased the PGC-1α protein levels in N2a cells (Fig. 4A,B). Additionally, to determine whether FG-4592 treatment was accompanied by functional changes in cellular metabolism, ATP production and a real-time analysis of mitochondrial respiration under various conditions were compared (Fig. 4C). The results demonstrated that basal respiration (Fig. 4D), ATP production (Fig. 4E), spare respiration capacity (Fig. 4F), and maximal respiration (Fig. 4G) in these neurons were significantly decreased by cumate treatment, suggesting that mitochondrial dysfunction is related to cumate-induced α-Syn. FG-4592 clearly ameliorated α-Syn-induced mitochondrial dysfunction (Fig. 4C–G). Nevertheless, the induction of PGC-1α by co-treated cumate and FG-4592 was not detected in α-Syn-N2a cells (data not shown). These findings suggest that FG-4592 may regulate mitochondrial biogenesis and respiration as well as the cell defense system against ROS possibly via HO-1 induction.

## Discussion

The current study’s goal was to identify whether FG-4592 had a neuroprotective effect against α-Syn-induced neurotoxicity in α-Syn-N2a cells, a cellular model of PD. We showed that FG-4592 induced significant neuroprotective effects. Nevertheless, FG-4592 had no effect on the amount of α-Syn protein in α-Syn-N2a cells. This protection is strongly correlated with the induction of HO-1 protein. Additionally, FG-4592 regulates mitochondrial biogenesis, and respiration and also the cell defense system against ROS. These results recognize FG-4592 as a novel neuroprotective agent against α-Syn-mediated neurotoxicity.

Synucleinopathies, a subset of neurodegenerative diseases that includes PD, dementia with Lewy bodies, and multiple system atrophy, are characterized by α-Syn-rich aggregates presence. The α-Syn protein functions centrally in the pathogenesis of these diseases. Genetic studies have verified that mutations and multiplications of the gene *SNCA* (https://www.ncbi.nlm.nih.gov/gene/6622) on chromosome 4q21–23, encoding α-Syn, result in early-onset familial PD, with widespread deposition of α-Syn aggregates in the brain^17^. There shows that under normal conditions, α-Syn is bound to mitochondria and may also be found in the mitochondrial lumen^17, 18^. Mitochondrial dysfunction is one of the first events in the PD brain, making the interaction between α-Syn and mitochondrial function critical in PD. An abundance of data demonstrated that overexpression of α-Syn is adequate to cause mitochondrial dysfunction and subsequent increased neuronal death^17, 19^. The mutant α-Syn (A53T) cellular environment in hiPSC-derived neurons, with intracellular aggregates, and oxidative stress together, caused accelerated oligomerization of exogenously added α-Syn. Finally, the presence of A53T oligomers was adequate to trigger mitochondrial dysfunction and result in cell death, thus corroborating the hypothesis that the mitochondria are a key mediator of α-Syn pathology. Hence, the removal of one of the primary drivers of mitochondrial dysfunction and oligomerization, ROS, could prevent cellular pathology in the mutant α-Syn (A53T) model^15^. In the current study, ROS was eliminated by HO-1 stimulated by FG-4592, and subsequently averted α-Syn-mediated cell death. Also, FG-4592 enhanced mitochondrial biogenesis and respiration in α-Syn-N2a cells. These results imply that FG-4592 may possess novel therapeutic potential for Synucleinopathies including PD.

The autophagy pathway closely partakes in the degradation of α-Syn protein. Disrupted protein homeostasis linked to the accumulation of α-Syn microaggregates in DA neurons is a constantly observed hallmark of PD^20^. Previously, it has been reported that FG-4592 induced autophagy in both PC12 cells obtained from a transplantable rat pheochromocytoma and primary cortical rat neurons^21^. FG-4592 also stimulated autophagy levels to suppress an in vitro ischemia model^22^. In the current investigation, we examined whether FG-4592 induced autophagy in α-Syn-N2a cells. Unfortunately, FG-4592 did not trigger autophagy in our experimental conditions (data not shown). Indeed, FG-4592 did not affect the amount of α-Syn protein in α-Syn-N2a cells that had been exposed to cumate for 48 h. A previous study using α-Syn-induced PD models, protective effects on such as inflammation without changing α-Syn level^23^, however this inconsistency in experimental results may be owing to factors such as cell origin, the gene expression system, and the experimental system. Although we could not explain this inconsistency, also considering other rotenone or MPTP-induced PD model, protection of mitochondrial and ROS toxicity is important therapeutic strategy for PD. Recently, the benefits of patient-derived iPS cells as a model for PD have been demonstrated^24^. Hence, studies under conditions closer to the pathological conditions of PD, such as using iPS cells, are required.

It has been noted that HIF-1α expression and its downstream targets in the SNpc of the PD brain are linked to the development of PD^7, 9^. For instance, HIF-1α can directly influence the expression of two PD-related genes, *LRRK2*, and *ATP13A2*, through the HREs in their promoters^9^. ATP13A2 provides protection against genetic and environmental risk factors for PD, whereas ATP13A2 loss compromises lysosomes^25^. As previously described, HIF-1α has various biological and pharmacological properties that function against many diseases, including neurodegenerative diseases such as PD. In the current research, FG-4592 lowered the production of ROS, induced the expression HO-1 and suppressed α-Syn-induced neurotoxicity. Although we could not determine direct relationship between HO-1 and mitochondrial dysfunction, the previous studies showed that induction of HO-1 expression protect mitochondrial dysfunction^26, 27^. In addition, FG-4592 may have neuroprotective effects against α-Syn-associated cell death through a multifaceted gene expression mechanism of action. Nrf2 is also well known as a major transcription factor to induce HO-1. Previous studies showed that increases of HIF-1α by FG-4592 caused Nrf2 activation^28^. The ARE-reporter assay was performed to determine whether Nrf2-ARE response pathway could be activated by FG-4592 treatment, however the level of reporter activity was not changed by FG-4592 (Supplementary Fig. 5). This study could not show Nrf2 activity induced by FG-4592. The different results between this study and the previous study could be possible due to the difference of experimental system or condition such as time of FG-4592 treatment. Various studies have shown that treatment with monoclonal antibodies could attenuate the propagation of pathology in preclinical models of PD^29–31^. Therefore, the efficacy of both antibody therapies has been limited^32, 33^. PD is a multifactorial disease incorporating a network of cellular pathways. Drugs having pleiotropic effects may practically be more effective than drugs with a single effect for PD patients. In the future, multiple agents including cocktail therapy employing antibodies and agents may be effective for PD. In the present research, FG-4592 did not change the amount of α-Syn protein. Conversely, it may be a candidate for one of the clinically therapeutic agents for PD, in combination with an appropriate antibody against α-Syn.

To investigate the molecular regulation of mitochondrial function by FG-4592, we evaluated the expression of PGC-1α, a transcriptional co-activator that regulates mitochondrial biogenesis and respiration in addition to the cell defense system against ROS^11, 34^. In the present research, FG-4592 increased the expression levels of PGC-1α in N2a cells. In contrast, in α-Syn-N2a cells, co-treated with cumate, and FG-4592 did not alter the PGC-1α protein level. In α-Syn-N2a cells, the expression level of PGC-1α was significantly decreased with the suppression of α-Syn by cumate and was not resolved by co-treatment with FG-4592. In these cells, the contribution of PGC-1α to the enhancement in mitochondrial function by FG-4592 may be limited. Alternatively, there may be another molecular pathway for the mitochondrial function-improving impacts of FG-4592 in the experimental conditions.

In conclusion, FG-4592 considerably suppressed α-Syn-induced neurotoxicity. The protective mechanism of FG-4592 determined in this research was the improvement of mitochondrial biosynthesis and respiration besides cellular defense systems against ROS possibly via HO-1 induction. Although other antioxidant should induce the same effects via similar molecular pathway such as HIF-1α/HO-1 pathway^35^, FG-4592 is a first-in-class orally active drug already approved for the treatment of renal anemia. Although there is possibility that FG-4592 might act by buffering ROS directly, there are no reports which suggested the direct effects of FG-4592 on ROS productions so far. As FG-4592 has numerous neuroprotective effects against α-Syn and also in regards to drug repositioning, FG-4592 may possess novel therapeutic potential for PD.

## Methods

### Cell culture

N2a cells used in this study was purchased from European Collection of Authenticated Cell Cultures. N2a cells was regulated in Dulbecco’s modified Eagle medium (DMEM, Wako Pure Chemical Industries, Ltd.) containing 10% (v/v) fetal bovine serum (FBS; Thermo Fisher Inc.) under a humidified atmosphere of 5% CO_2_ at 37 °C. α-Syn-N2a cells were prepared as previously described^12^. The cells were passaged by trypsinization every 3–4 days.

### CCK-8 assay

N2a cells and α-Syn-N2a cells were seeded at 5 × 10^4^ cells/mL in 24-well plates in DMEM comprising 10% FBS. The number of live cells was estimated utilizing a Cell Counting Kit-8 according to the manufacturer’s protocol (Wako Pure Chemical Industries Ltd.) as previously stated^12^. N2a cells were treated with 10, 20, 30, 50, and 100 µM FG-4592 (Cayman CHEMICAL). α-Syn-N2a cells were treated with 50 µg/mL of cumate in the presence or absence of 10, 30, or 50 µM FG-4592 and 3 µM Zinc protoporphyrin for 48 or 72 h. The number of live cells was projected by the Cell Counting Kit-8, following the manufacturer’s instructions (Wako Pure Chemical Industries Ltd.). Briefly, the reagent was included in the wells and the plate was incubated at 37 °C for 2 h. Cell viability was computed through the detection of the optical density of formazan at 450 nm utilizing Varioskan LUX (Thermo Fisher Scientific). A 600 nm wavelength was employed as a reference.

### Immunoblotting

Immunoblotting was carried out as described previously^36^. After treatment, the cells were lysed with RIPA buffer (50 mM Tris–HCl (pH7.5), 150 mM NaCl, 1% NP-40, 0.5% deoxycholic acid, 1% SDS, protease inhibitor cocktail) or 2% SDS buffer (50 mM Tris–HCl, 2% SDS, 10% glycerol); then the lysates were sonicated and centrifuged at 20,600×*g* at 4 °C for 20 min. The supernatant protein sample was acquired. Protein concentrations were measured utilizing a BCA protein assay kit (FUJIFILM Wako Pure Chemical Corporation) with bovine serum albumin as a standard. Lysates were mixed with a sample buffer containing 10% 2-mercaptoethanol, and subjected to 10% SDS–polyacrylamide gel electrophoresis (SDS-PAGE). SDS-PAGE was carried out under a constant voltage of 200 V at room temperature for 50 min. The separated proteins in polyacrylamide gel were transferred to a PVDF membrane in a transfer buffer (0.3% Tris, 1.44% glycine, 20% methanol) under a constant voltage of 100 V at 4 °C for 60 min. The membranes were incubated with 5% Skim milk (nacalai tesque) or Bloking One (nacalai tesque) for 60 min, and then with the following primary antibodies overnight: mouse monoclonal antibodies against β-actin (1:2000, Sigma), HIF-1α (1:1000, Novus Biologicals, Inc), PGC-1α (1:5000, proteintech), rabbit polyclonal antibody against HO-1 (1:2500, Enzo), and rabbit monoclonal antibody against α-synuclein (1:2500, abcam). The membrane was incubated after the primary antibody reaction in the secondary antibody (goat anti-rabbit antibody conjugated with HRP (1:2500, Sigma) or goat anti-mouse HRP antibody conjugated with HRP (1:5000, Sigma). The membrane was incubated in ECL prime (GE Healthcare) to produce chemiluminescence from HRP antibodies. The chemiluminescence was observed by a Fusion System (Vilber-Lourmat). The band density was quantified using ImageJ.

### RNA preparation and qRT-PCR

Reverse transcription was conducted utilizing the ReverTra Ace qPCR RT Master Mix, following the manufacturer’s instructions (TOYOBO) as previously described^37^. qRT-PCR was carried out using SYBR Green on a QuantStudio1 Real-Time PCR System, according to the manufacturer’s instructions (Life Technologies). The sequences of gene-specific primer sets are demonstrated in Table 1. The expression levels of mRNA were normalized to the expression levels of *Gapdh* mRNA.

**Table 1:** Primer pairs used for qRT-PCR.

	Forward	Reverse
*Hmox1*	5′tcgaatgaacactctggagatgaca3′	5′actctggtctttgtgttcctctgt3′
*Nqo1*	5′ggtttacagcattggccacact3′	5′aacaggctgcttggagcaaa3′
*Gclm*	5′tcacaatgacccgaaagaactg3′	5′acccaatcctgggcttcaat3′
*Gapdh*	5′tgcatcctgcaccacca3′	5′tcacgccacagctttcca3′

### Dual-luciferase reporter assay

The cells were transfected with the basic vectors lacking promoter, pGL4.42[luc2P/HRE/Hygro] comprising four copies of the hypoxia response element (HRE) that drives transcription of the luciferase reporter gene luc2P (*Photinus pyralis*), pGL4.37[luc2P/ARE/Hygro] comprising four copies of an antioxidant response element (ARE) that drives transcription of the luciferase reporter gene luc2P, and the hRluc (*Renilla reniformis*) luciferase vector pGL4.74[hRluc/TK] was cotransfected to normalize for the transfection efficiency as previously reported^38^. At 24 h following transfection to N2a cells in each vector, the cells were treated with 10, 30, or 100 µM FG-4592 for 2 h. Cells were harvested hours later after treatment with 1 × passive lysis buffer (Promega). Firefly luciferase activities and Renilla luciferase activities were assayed employing the Dual-luciferase reporter assay system (Promega). The firefly luciferase activity was normalized following hRluc luciferase activity and expressed as relative luciferase units to highlight the promoter activity.

### ROS detection

α-Syn-N2a cells were seeded at 1.5 × 10^5^ cells/compartment in CELLview cell culture dishes (Greiner Bio-one). The cells were treated with 50 µg/mL of cumate in the presence or absence of 30 µM FG-4592. At 48 h following the treatment, ROS were stained with OxiORANGE (Goryokayaku), and nuclei were stained with Hoechst 33342 (Thermo Fisher Scientific), following the manufacturer’s instructions (Goryokayaku) as previously described^12^. Fluorescent microscopy images were obtained with a confocal fluorescence microscope (LSM700, Carl Zeiss). Image analysis computed the fluorescence intensities in each image utilizing ImageJ (threshold criteria: 30-255 and then brightness measurement) and obtained the ratio by the number of cells stained with Hoechst. This quantitative analysis was applied to all cells in 5 images per experimental group.

### Seahorse XF mito stress test

Oxygen consumption rates (OCRs) were quantified using the XF Extracellular Flux Analyzer (Seahorse Bioscience). α-Syn-N2a cells were seeded at a density of 5.0 × 10^4^ cells/well employing a Seahorse plate coated with 0.2% gelatin and cultured overnight. Cells were equilibrated with XF Base media (Seahorse, catalog number 102353-100) at 37 °C for 1 h in an incubator lacking CO_2_. Mitochondrial stress was measured using an Agilent Seahorse XF Cell Mito Stress Test Kit (Seahorse) based on the manufacturer’s protocol followed by sequential treatments with oligomycin (1.4 μM), FCCP (1.0 mM), and rotenone/antimycinA (0.5 μM) (Seahorse).

### Statistical analysis

Data are expressed as the mean ± standard error of the mean (SEM). Significance was determined employing the Student’s t-test or analysis of variance. Further statistical analysis for post hoc comparisons was conducted utilizing the Bonferroni/Dunn test (StatView, Abacus). p-values of less than 0.05 were considered to be statistically significant.

### Supplementary Information

Supplementary Figures.

## Data Availability

The datasets used and/or analyzed during the current study are available from the corresponding author on reasonable request.
