# Supplementary material for: The neuroprotective effects of FG-4592, a hypoxia-inducible factor-prolyl hydroxylase inhibitor, against oxidative stress induced by alpha-synuclein in N2a cells
Source: Sci Rep. 2023 Sep 20;13:15629. doi: 10.1038/s41598-023-42903-7 (PMC10511692; doi:10.1038/s41598-023-42903-7)
Supplement: Supplementary file 1 — Supplementary Figures. [file 41598_2023_42903_MOESM1_ESM.docx]

**Supplementary Information**

**The neuroprotective effects of FG-4592, a hypoxia-inducible factor prolyl-hydroxylase inhibitor, against oxidative stress induced by alpha-synuclein in N2a cells**

**Ayaka Fujimaki^1^, Kazuki Ohuchi^1^, Shinnosuke Takizawa^1^, Takanori Murakami^1^, Hisaka Kurita^1^, Isao Hozumi^1^, Xiaopeng Wen^2^, Yoshihisa Kitamura^2^, Zhiliang Wu^3^, Yoichi Maekawa^3,4,5^, and Masatoshi Inden^1^***

^1^Laboratory of Medical Therapeutics and Molecular Therapeutics, Gifu Pharmaceutical University, Gifu, 501-1196, Japan.

^2^Laboratory of Pharmacology and Neurobiology, College of Pharmaceutical Sciences, Ritsumeikan University, Shiga, 525-8577, Japan.

^3^Department of Parasitology and Infectious Diseases, Gifu University Graduate School of Medicine, Gifu, 501-1194, Japan.

^4^Division of Preemptive Food Research, Preemptive Food Research Center (PFRC), ^5^Division of Animal Medical Science, Center for One Medicine Innovative Translational Research (COMIT), Gifu University Institute for Advanced Science (GUIAS), Gifu, 501-1194, Japan.

*Corresponding Authors: inden@gifu-pu.ac.jp



**(a)** Figure 1C α-synuclein (14 kDa)

**α-synuclein**

14 kDa

48 kDa

35 kDa

25 kDa

**(b)** Figure 1C β-actin (42 kDa)

**

**

**β-actin**

42 kDa

35 kDa

48 kDa

**

**Original full-length image

Full-length image after gaining contrast

**

**

Full-length image with bright field

**

**

**Supplementary Figure 1.** (a) Whole representative western blot of α-Syn; (b) Whole representative western blot of β-actin. Original full-length image, full-length image after gaining contrast and full-length image with bright field are presented for western blot of β-actin. The portion surrounded by a red square is presented in Fig. 1C.

1. Figure 2B HIF-1α (100~120 kDa)





**HIF-1α**

100~120 kDa

135 kDa

100 kDa

1.

Figure 2B β-actin (42 kDa)

**β-actin**

42 kDa

48 kDa

35 kDa

Original full-length image





**

**Full-length image after gaining contrast

Full-length image with bright field

**

**

1. Figure 2H HO-1 (32 kDa)

**

**

25 kDa

35 kDa

**HO-1**

32 kDa

63 kDa

48 kDa

1. Figure 2H β-actin (42 kDa)





35 kDa

**β-actin**

42 kDa

48 kDa



Original full-length image



Full-length image after gaining contrast



Full-length image with bright field

**Supplementary Figure 2.** (a) Whole representative western blot of HIF-1α; (b) Whole representative western blot of β-actin; (c) Whole representative western blot of HO-1; (d) Whole representative western blot of β-actin. Original full-length image, full-length image after gaining contrast and full-length image with bright field are presented for western blot of β-actin. The portion surrounded by a red square is presented in Fig. 2B and Fig. 2H.

1. Figure 3C HO-1 (32 kDa)





**HO-1**

32 kDa

63 kDa

25 kDa

35 kDa

48 kDa



Original full-length image



Full-length image after gaining contrast



Full-length image with bright field



**(b)** Figure 3C β-actin (42 kDa)

35 kDa

48 kDa

**β-actin**

42 kDa



Original full-length image



Full-length image after gaining contrast



Full-length image with bright field

**Supplementary Figure 3.** (a) Whole representative western blot of HO-1; (b) Whole representative western blot of β-actin. Original full-length image, full-length image after gaining contrast and full-length image with bright field are presented for western blot of HO-1 and β-actin. The portion surrounded by a red square is presented in Fig. 3C.

1. Figure 4A PGC-1α (100 kDa)





**PGC-1α**100 kDa

135 kDa

75 kDa

100 kDa



Original full-length image



Full-length image after gaining contrast



Full-length image with bright field



**(b)** Figure 4A β-actin (42 kDa)

**β-actin**

42 kDa

35 kDa

48 kDa



Original full-length image



Full-length image after gaining contrast



Full-length image with bright field

**Supplementary Figure 4.** (a) Whole representative western blot of PGC-1α; (b) Whole representative western blot of β-actin. Original full-length image, full-length image after gaining contrast and full-length image with bright field are presented for western blot of PGC-1α and β-actin. The portion surrounded by a red square is presented in Fig. 4A.


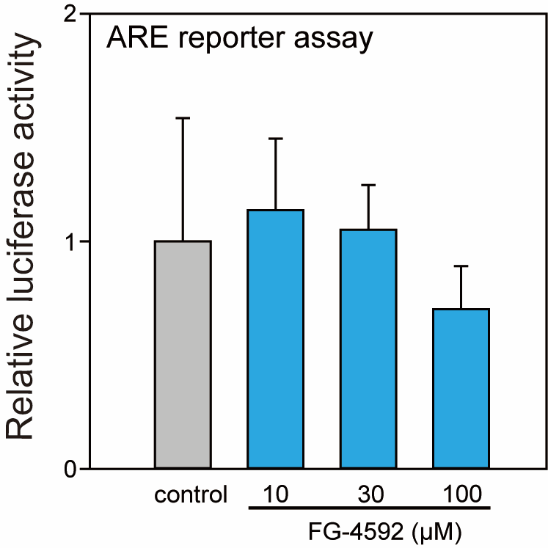


**Supplementary Figure 5.** Results of ARE luciferase assays.

**
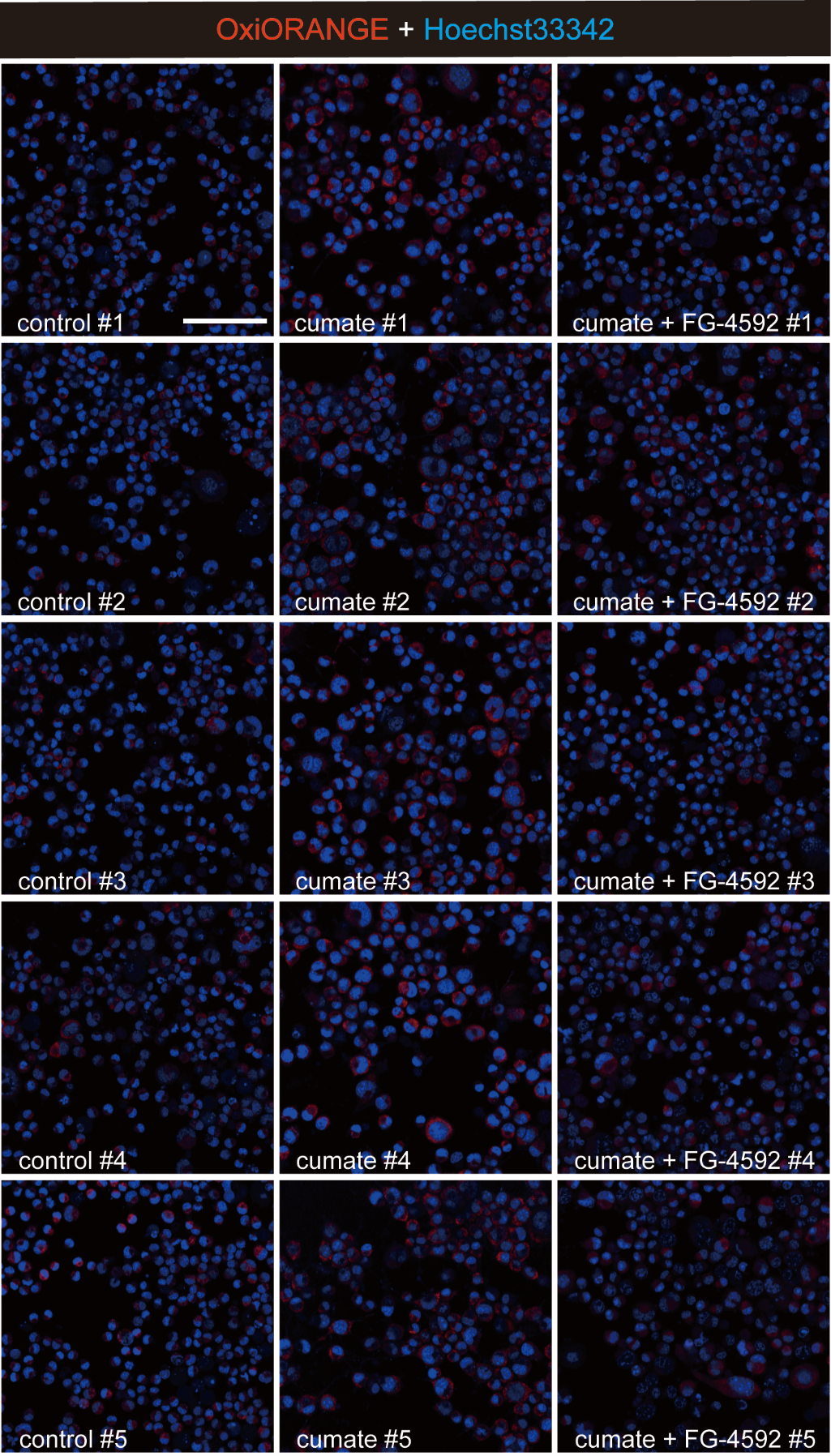
**

**Supplementary Figure 6.** All individual image data used to calculate fluorescence intensity of figure 3B. The images of #1 in each experimental group were presented in figure 3A.
